# Evaluation of myosin VI, E-cadherin and beta-catenin immunostaining in renal cell carcinoma

**DOI:** 10.1186/1756-9966-29-2

**Published:** 2010-01-14

**Authors:** Hanna Ronkainen, Saila Kauppila, Pasi Hirvikoski, Markku H Vaarala

**Affiliations:** 1 Department of Surgery, PO Box 21, Oulu University Hospital, FIN-90029 Oulu, Finland; 2 Department of Pathology, PO Box 50, Oulu University Hospital, FIN-90029 Oulu, Finland

## Abstract

**Background:**

Renal cell carcinoma (RCC) is a cancer of increasing incidence and mortality. Currently, there are no immunohistochemical prognostic markers for RCCs in routine use. The aim of this study was to examine for the first time the immunostaining of myosin VI in RCCs as well as its association with E-cadherin and beta-catenin immunostaining and the prognostic significance of these markers in RCCs.

**Methods:**

Our study population consisted of 152 patients who underwent surgery for RCCs between 1990 and 1999. The tumours were examined with three immunohistochemical markers: myosin VI, E-cadherin and beta-catenin.

**Results:**

The immunostaining for cytoplasmic myosin VI was common (72%). One-third of the tumours were immunopositive for nuclear myosin VI. Cytoplasmic myosin VI immunopositivity and nuclear beta-catenin immunostaining were associated with lower Fuhrman grades (*p *= 0.04 and *p *= 0.005, respectively), but not stages. There was no significant association between myosin VI immunostaining and the histological subtype of RCC. Nuclear myosin VI was associated with the nuclear expression of beta-catenin. A direct association could also be proven between membranous E-cadherin and cytoplasmic beta-catenin. Cytoplasmic myosin VI immunostaining was a marker of poorer prognosis in multivariate Cox regression model adjusted with stage and Fuhrman grade with hazard ratio 2.4 (95% confidence interval 1.1 to 5.0 with *p *= 0.024).

**Conclusions:**

Cytoplasmic myosin VI immunopositivity and nuclear beta-catenin immunostaining were associated with lower Fuhrman grades, and there was a strong positive relationship between E-cadherin immunostaining and beta-catenin immunostaining in RCCs. Cytoplasmic myosin VI immunostaining was associated with poorer prognosis in RCCs.

## Background

Renal cell carcinoma (RCC) is a cancer of increasing incidence and mortality [1]. By diagnosis, up to one-third of patients already have a metastasised disease and half of the remaining patients will suffer a recurrence after curative treatment [2]. The behaviour of RCC can be difficult to predict even though there are many well-known prognostic factors for the disease. Myosins are a large family of molecular motor proteins and their immunoexpression has previously been demonstrated in variety of epithelial cancers including RCCs [3-5]. Myosin VI is one of the so-called unconventional myosins that moves in a reverse direction when compared to the other known myosins, i.e. it moves from the plasma membrane into the cell and away from the surface of internal organelles such as the Golgi complex. Myosin VI plays a role both in transporting and anchoring the cells and takes part in a wide range of cellular processes such as endocytosis, exocytosis, cell migration, cell division and cytokinesis [3,6]. Furthermore, myosin VI is linked to E-cadherin and beta-catenin in ovarian cancer [7].

One of the key processes in developing metastasised disease is a loss of cellular adhesion [8]. E-cadherin, a member of the adhesion molecule family of cadherins, mediates predominantly cell-cell adhesion in epithelium and epithelial tumours. It is a tumour suppressor, the loss of which is known to worsen the prognosis of many cancers. The whole function of E-cadherin is not yet well understood but it might influence the transcription of target genes [9-15]. Transmembranic glycoprotein E-cadherin interacts with the cytoskeleton via intracellular proteins named catenins. Cell-cell cohesion can be damaged by the loss of E-cadherin expression or changes in catenin expression, which leads to the loss of cadherin function. The cadherin-catenin complex also influences migration and modifies cell growth and the survival of neoplastic cells [8]. In addition, beta-catenin, a member of the catenin family, participates in signal transduction [16,17].

There are no current immunohistochemical prognostic markers for RCCs in routine use. In this era of new treatment possibilities there remains a need for better prognostic tools to plan the treatment and follow-up of RCC patients. The purpose of this study was to examine for the first time the immunostaining of myosin VI in RCCs and to investigate the prognostic potential of immunostaining myosin VI, E-cadherin and beta-catenin in RCCs.

## Methods

### Patients

The study population has been described in detail earlier [18]. Briefly, the retrospective study population consisted of 152 patients who underwent surgery for RCCs between 1990 and 1999 at the Oulu University Hospital in Finland. Seven patients (5%) were operated by resection and 145 (95%) by radical nephrectomy. The patients' follow-up details were collected from patient records. Follow-up was completed in all cases. The research plan was approved by the local ethical board. The stage of the tumours was assigned using the TNM (tumour-node-metastasis) staging of RCCs [19].

### Tumour samples

The tumour samples were fixed in 10% buffered formalin and embedded in paraffin. Histological diagnosis was confirmed by reviewing haematoxylin and eosin (H & E)-stained original sections. The tumours were reclassified and graded according to the WHO classification [20]. The most representative block was selected to reconstruct a multitissue block, which was used for immunohistochemistry.

### Immunostaining procedure

The immunoexpression of myosin VI, E-cadherin and beta-catenin was analysed using monoclonal antibodies. The antibodies used in the study were monoclonal anti-myosin VI (Sigma, St. Louis, MO, USA) in a dilution of 1:250, mouse anti-E-cadherin (Zymed Laboratories, San Francisco, CA, USA) in a dilution of 1:300 and anti-beta-catenin (BD Biosciences, San Jose, CA, USA) in a dilution of 1:200. For antigen retrieval, the sections were incubated in 0.01 M citrate buffer (pH 6) twice for 5 min and boiled in a microwave oven to enhance immunoreactivity. The sections were cooled for 15 min in 0.05 M Tris buffered saline (TBS) (pH 7.5) and washed twice in PBS. Endogenous peroxidise activity was eliminated by incubation in 5% hydrogen peroxide and absolute methanol. Bound antibodies were visualised using an EnVision+ System-HRP (DakoCytomation, Glostrup, Denmark).

### Immunohistochemical evaluation of the markers

Immunohistochemical staining was evaluated simultaneously by three observers (HR, PH and SK) and a consensus was reached. Immunostaining for cytoplasmic myosin VI and membranous E-cadherin was classified as follows: negative and weak positive were considered negative and moderate and strong positive were considered positive. Immunostaining was classified negative and positive for nuclear myosin VI, E-cadherin and beta-catein as well as cytoplasmic beta-catein. The result was considered positive when any staining was detected.

### Statistical analyses

SPSS for Windows 15 (Chicago, IL, USA) was used for statistical analyses. The chi-squared test or Fisher's exact test was used to study associations between different variables. Survival was analysed with the Kaplan-Meier curve and significance with the log rank test. The Cox regression multivariate model was used for multivariate analysis using Fuhrman grade, stage, tumour diameter, age or gender as adjusting factors.

## Results

### Patient demographics and staining correlation with clinical parameters

At the time of diagnosis, the median age of patients was 63 years (range 29-86 years). Seventy-seven (51%) patients were women and 75 (49%) men. The median follow-up time was 90 months (range 0-209 months). During follow-up, 44 (29%) patients died because of RCCs, 40 (26%) died of other causes and 68 (45%) patients were still alive. The distribution of tumour classes (TNM classification), clinical stages, tumour grades and the histological subtype of the RCC in comparison to the immunostaining pattern for myosin VI, beta-catenin and E-cadherin are described in Table 1, Table 2 and Table 3, respectively.

**Table 1:** Associations between immunostaining for myosin VI and tumour class, stage, grade and histological subtype of RCC.

	Cytoplasmic myosin VI		Nuclear myosin VI	
	positive	negative	positive	negative
Tumour class (T)				

1 (n = 71)	54 (76%)	17 (24%)	25 (35%)	46 (65%)
2 (n = 11)	6 (55%)	5 (45%)	3 (27%)	8 (73%)
3 (n = 57)	41 (72%)	16 (28%)	20 (35%)	37 (65%)
4 (n = 6)	3 (50%)	3 (50%)	3 (50%)	3 (50%)
				
Stage				

I (n = 66)	50 (76%)	16 (24%)	23 (35%)	43 (65%)
II (n = 11)	6 (55%)	5 (45%)	3 (27%)	8 (73%)
III (n = 49)	35 (71%)	14 (29%)	19 (39%)	30 (61%)
IV (n = 19)	13 (68%)	6 (32%)	6 (32%)	13 (68%)
				
Grade				

I (n = 5)	5 (100%)	0 (0%)	1 (20%)	4 (80%)
II (n = 79)	59 (75%)	20 (25%)	31 (39%)	48 (61%)
III (n = 38)	28 (74%)	10 (26%)	10 (26%)	28 (74%)
IV (n = 21)	10 (48%)	11 (52%)	8 (38%)	13 (62%)
				
Histological subtype of RCC				

clear cell (n = 128)	89 (70%)	39 (30%)	46 (36%)	82 (64%)
papillary (n = 10)	9 (90%)	1 (10%)	2 (20%)	8 (80%)
chromophobic (n = 5)	4 (80%)	1 (20%)	2 (40%)	3 (60%)
undifferentiated (n = 2)	2 (100%)	0 (0%)	1 (50%)	1 (50%)

Number of patients with different characteristics and respective cytoplasmic and nuclear myosin VI immunostaining are presented.

**Table 2:** Associations between immunostaining for beta-catenin and tumour class, stage, grade and histological subtype of RCC.

	Cytoplasmic beta-catenin		Nuclear beta-catenin	
	positive	negative	positive	negative
Tumour class (T)				

1 (n = 71)	5 (7%)	66 (93%)	36 (51%)	35 (49%)
2 (n = 11)	2 (18%)	9 (82%)	2 (18%)	9 (82%)
3 (n = 59)	6 (10%)	53 (90%)	24 (41%)	35 (59%)
4 (n = 6)	0 (0%)	6 (100%)	3 (50%)	3 (50%)
				
Stage				

I (n = 66)	5 (8%)	61 (92%)	34 (52%)	32 (48%)
II (n = 11)	2 (18%)	9 (82%)	2 (18%)	9 (82%)
III (n = 51)	5 (10%)	46 (90%)	21 (41%)	30 (59%)
IV (n = 19)	1 (5%)	18 (95%)	8 (42%)	11 (58%)
				
Grade				

I (n = 5)	0 (0%)	5 (100%)	2 (40%)	3 (60%)
II (n = 80)	6 (7%)	74 (93%)	46 (58%)	34 (42%)
III (n = 38)	2 (5%)	36 (95%)	9 (24%)	29 (76%)
IV (n = 22)	4 (18%)	18 (82%)	8 (36%)	14 (64%)
				
Histological subtype of RCC				

clear cell (n = 130)	10 (8%)	120 (92%)	58 (45%)	72 (55%)
papillary (n = 10)	1 (10%)	9 (90%)	4 (40%)	6 (60%)
chromophobic (n = 5)	1 (20%)	4 (80%)	3 (60%)	2 (40%)
undifferentiated (n = 2)	1 (50%)	1 (50%)	0 (0%)	2 (100%)

Number of patients with different characteristics and respective cytoplasmic and nuclear beta-catenin immunostaining are presented.

**Table 3:** Associations between immunostaining for E-cadherin and tumour class, stage, grade and histological subtype of RCC.

	Membranous E-cadherin		Nuclear E-cadherin	
	positive	negative	positive	negative
Tumour class (T)				

1 (n = 72)	7 (10%)	65 (90%)	34 (47%)	38 (53%)
2 (n = 11)	1 (9%)	10 (91%)	5 (45%)	6 (55%)
3 (n = 59)	5 (8%)	54 (92%)	17 (29%)	42 (71%)
4 (n = 6)	1 (17%)	5 (83%)	3 (50%)	3 (50%)
				
Stage				

I (n = 67)	6 (9%)	61 (91%)	32 (48%)	35 (52%)
II (n = 11)	1 (9%)	10 (91%)	5 (45%)	6 (55%)
III (n = 51)	5 (10%)	46 (90%)	13 (25%)	38 (75%)
IV (n = 19)	2 (10%)	17 (90%)	9 (47%)	10 (53%)
				
Grade				

I (n = 5)	0 (0%)	5 (100%)	1 (20%)	4 (80%)
II (n = 81)	4 (5%)	77 (95%)	38 (47%)	43 (53%)
III (n = 38)	5 (13%)	33 (87%)	14 (37%)	24 (63%)
IV (n = 22)	3 (14%)	19 (86%)	6 (27%)	16 (73%)
				
Histological subtype of RCC				

clear cell (n = 131)	8 (6%)	123 (94%)	52 (40%)	79 (60%)
papillary (n = 10)	0 (0%)	10 (100%)	6 (60%)	4 (40%)
chromophobic (n = 5)	4 (80%)	1 (20%)	1 (20%)	4 (80%)
undifferentiated (n = 2)	2 (100%)	0 (0%)	0 (0%)	2 (100%)

Number of patients with different characteristics and respective membranous and nuclear E-cadherin immunostaining are presented.

### Myosin VI immunostaining in RCCs

**Figure 1:**
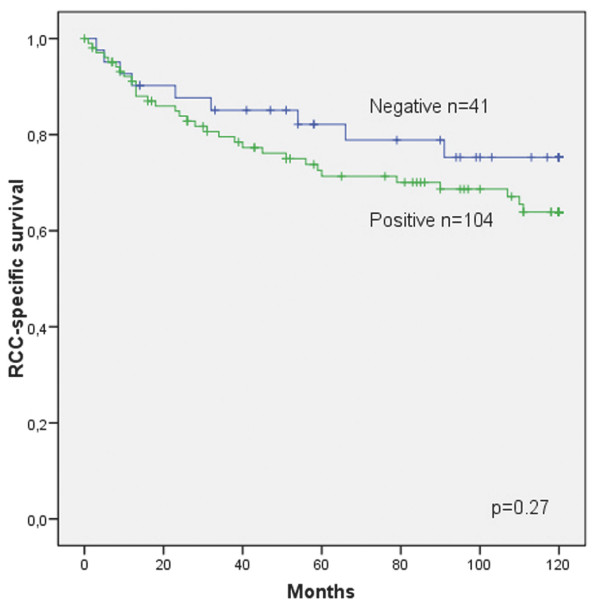
**Cytoplasmic myosin VI as a prognostic factor in ten-year RCC-specific survival**. Kaplan-Meier curve of 145 patients. *p *= 0.27.

Cytoplasmic myosin VI was positive in 104 (72%) and negative in 41 (28%) cases. Cytoplasmic myosin VI immunopositivity was associated with lower Fuhrman grades (*p *= 0.04) but not stages (Table 1). In univariate survival analysis, the patients with RCCs expressing cytoplasmic myosin VI had a tendency for lower RCC-specific survival, but this was not significant (*p *= 0.27 for ten-year RCC-specific survival) (Figure 1, Table 4). However, adjusting Cox regression model with the known prognostic factors of RCCs, stage and Fuhrman grade, cytoplasmic myosin VI immunostaining was a prognostic marker with hazard ratio (HR) 2.4 (95% confidence interval (CI) 1.1 to 5.0 with *p *= 0.024). When tumour diameter was retained in the model, HR for cytoplasmic myosin VI was 3.5 (95% CI 1.5 to 7.9) with *p *= 0.003. Further adjusting the model with age or gender, which were not statistically significant factors, the HR for cytoplasmic myosin VI was 2.4 (*p *= 0.025) and 2.4 (*p *= 0.025), respectively. The mean survival times for subjects with Fuhrman grade II cytoplasmic myosin VI immunonegative and immunopositive tumours died of RCC during follow-up were 101 (standard deviation (SD) ± 71) and 52 (SD ± 47) months, respectively. None of the patients with Fuhrman grade I tumours died of RCC during the follow-up. Immunostaining for nuclear myosin VI was observed in 51 (35%) cases. Myosin VI immunostaining was not associated with the histological subtype of RCCs (Table 1). Nuclear immunostaining for myosin VI was not a prognostic factor in RCC-specific survival (*p *= 0.9) (Table 4) and did not correlate with Fuhrman grades or stages (Table 1).

**Table 4:** The RCC-specific mean survivals for myosin VI, E-cadherin and beta-catenin immunostaining.

Marker	Immunostaining result	Mean survival (months)	95% CI	p-value
Cytoplasmic myosin VI	negative	162	137-187	0.3
	positive	146	128-163	
				
Nuclear myosin VI	negative	151	134-169	0.9
	positive	141	118-164	
				
Membranous E-cadherin	negative	153	138-168	0.3
	positive	113	73-152	
				
Nuclear E-cadherin	negative	144	124-164	0.4
	positive	158	137-179	
				
Cytoplasmic beta-catenin	negative	152	137-167	0.5
	positive	128	81-174	
				
Nuclear beta-catenin	negative	143	124-163	0.3
	positive	157	136-178	

*P *values presented were produced with the log rank test. CI, confidence interval.

### Beta-catenin immunostaining in RCCs

Nuclear staining for beta-catenin was seen in 65 (44%) cases and cytoplasmic staining in 13 (9%). Nuclear beta-catenin immunoexpression was associated with lower Fuhrman grades (*p *= 0.005) but not stages (Table 2). Cytoplasmic staining for beta-catenin was not associated with stages or grades (Table 2). There was no relationship between the histological subtype of RCCs and immunoreactivity for beta-catenin. For RCC-specific survival beta-catenin immunoexpression had no prognostic significance (Table 4).

### E-cadherin immunostaining in RCCs

Membranous staining for E-cadherin was observed in 14 (9%) cases and nuclear staining in 59 (40%). Membranous staining for E-cadherin was associated with histological subtype (*p *< 0.001). It was more common in chromophobic and unclassified subtypes than in clear cell RCCs, whereas no positivity was observed in papillary subtypes (Table 3). Nuclear E-cadherin immunoexpression and the histological subtype of RCCs were unassociated (Table 3). Neither stage nor differentiation was associated with the E-cadherin staining pattern (Table 3). The nuclear or membranous expression of E-cadherin was not a prognostic factor for RCC-specific survival (Table 4).

### Association between myosin VI, beta-catenin and E-cadherin immunostaining in RCCs

Nuclear myosin VI was associated with beta-catenin immunostaining (*p *= 0.008). The relationship between nuclear myosin VI and E-cadherin and cytoplasmic myosin VI and membranous E-cadherin were not significant (*p *= 0.09 and *p *= 0.07, respectively). Nuclear staining patterns for E-cadherin and beta-catenin (*p *< 0.001) and membranous E-cadherin and cytoplasmic beta-catenin (*p *= 0.02) were associated with each other. The associations between E-cadherin, beta-catenin and myosin VI immunostaining are represented in Table 5.

**Table 5:** Association between immunostaining for myosin VI, E-cadherin and beta-catenin.

		Nuclear myosin VI		p-value
		negative	positive	
Nuclear beta-catenin	negative	59 (74%)	21 (26%)	
	positive	33 (52%)	30 (48%)	0.008
				
		Cytoplasmic myosin VI		
		Negative	positive	

Cytoplasmic beta-catenin	negative	38 (29%)	92 (71%)	
	positive	3 (23%)	10 (77%)	0.8*
				
		Nuclear myosin VI		
		negative	positive	

Nuclear E-cadherin	negative	61 (70%)	26 (30%)	
	positive	32 (56%)	25 (44%)	0.09
				
		Cytoplasmic myosin VI		
		negative	positive	

Membranous E-cadherin	negative	40 (31%)	90 (69%)	
	positive	1 (7%)	13 (93%)	0.07*
				
		Nuclear beta-catenin		
		negative	positive	

Nuclear E-cadherin	negative	66 (75%)	22 (25%)	
	positive	16 (27%)	43 (73%)	<0.001
				
		Cytoplasmic beta-catenin		
		negative	positive	

Membranous E-cadherin	negative	124 (93%)	9 (7%)	
	positive	10 (71%)	4 (29%)	0.02*

*P *values presented were produced with the chi-squared test or Fisher's exact test (*).

## Discussion

This was the first study characterising the expression of myosin VI in RCCs. Here, cytoplasmic myosin VI immunopositivity was associated with the lower Fuhrman grades of RCCs, but in multivariate Cox regression model it was also a marker of poorer prognosis. The immunoexpression of myosin VI has been demonstrated in prostatic adenocarcinoma [21,22]. There is also evidence that links myosin VI to the migration of human ovarian cancer cell lines [23]. In ovarian carcinomas, myosin VI expression has been associated with the aggressive behaviour of the tumour [24]. In our study, cytoplasmic myosin VI immunostaining was not a statistically significant prognostic factor according to log rank test. However, in multivariate Cox regression model adjusted with the known prognostic factors of RCCs, stage and Fuhrman grade, cytoplasmic myosin VI immunostaining was a prognostic marker for RCC specific survival. This means, that confounding factors affecting the results of log rank test were present, which could be reduced in Cox regression model. Noteworthy, the HR for cytoplasmic myosin VI immunostaining was increased also when tumour diameter, age or gender was retained to the model. Despite the association of cytoplasmic myosin VI immunopositivity with lower Fuhrman grades, the mean survival times for subjects with cytoplasmic myosin VI immunopositive Fuhrman grade II tumours died of RCC during follow-up were shorter in comparison with subjects with cytoplasmic myosin VI immunonegative tumours. So cytoplasmic myosin VI immunopositivity seems to have prognostic potential also within Fuhrman grade II tumours but not only within poorly differentiated tumours.

It has been reported that membranous beta-catenin immunoexpression is downregulated in conventional RCCs with low nuclear grades but higher in papillary and chromophobic carcinomas than conventional RCCs [25]. In our study, nuclear beta-catenin immunostaining was more frequently detected in cases with lower Fuhrman grades, but we found no prognostic significance of beta-catenin immunostaining in RCCs. Furthermore, we detected no differences in beta-catenin immunoexpression patterns between the different histological subtypes of RCCs.

According to our study, nuclear E-cadherin expression is neither an independent prognostic factor in RCC-specific survival nor associated with the nuclear grade of the tumour. Nuclear E-cadherin has previously been demonstrated to be associated with better prognosis of RCCs [15], and there has also been a reported downregulation of E-cadherin expression in clear cell RCCs [26]. In our study population, we could not prove the prognostic importance of E-cadherin that had previously been shown in smaller study populations and with shorter follow-up times. In previous studies, nuclear E-cadherin expression was detected only in clear cell RCCs [15]. In our study, some nuclear positivity was also demonstrated in papillary and chromophobic carcinomas.

According to our study, nuclear myosin VI is associated with beta-catenin but there is no relationship between myosin and E-cadherin in RCCs. Myosin VI is linked to E-cadherin and beta-catenin and participates in border cell migration where it stabilises the E-cadherin-beta-catenin cell adhesion complex [7]. Myosin VI is a cytoplasmic protein and the significance of nuclear myosin VI immunostaining is unknown. Beta-catenin, however, can be detected in the nucleus in various carcinomas [27-30]. Nuclear myosin VI could be a regulating factor for beta-catenin or a co-worker. The association between myosin VI and beta-catenin might also suggest that beta-catenin provides a molecular mechanism for signal transduction from the cytoplasm to the nucleus of the cell, thereby also influencing myosin VI gene expression. Beta-catenin plays a role in the *Wnt *(wingless type) pathway where the multiprotein destruction complex which involves APC (adenomatous polyposis coli) influences the phosphorylation and unphosphorylation of beta-catenin and has been demonstrated to lead to the transcription and expression of oncogenes such as c-myc and c-jun [16,17]. Beta-catenin has also been reported to be capable of regulating gene expression by the direct interaction with transcription factors such as LEF-1 (lymphoid enhancer-binding factor), providing a molecular mechanism for a signal transmission from cell-adhesion components to the nucleus [16]. In our study, we also found a positive relationship between nuclear E-cadherin and beta-catenin immunostaining, which has previously been detected in gastrointestinal carcinomas [15,31,32].

## Conclusions

Our findings were that in RCCs there is immunoexpression of myosin VI in cytoplasm and nucleus, and cytoplasmic myosin VI is an independent prognostic factor in RCC-specific survival. In the future, myosin VI may have use as a prognostic marker of RCCs. Cytoplasmic myosin VI immunopositivity and nuclear beta-catenin immunostaining were associated with lower Fuhrman grades but not stages. Nuclear myosin VI and beta-catenin immunoexpression are associated with each other. Nuclear E-cadherin and beta-catenin immunostaining patterns are also positively related together. The discrepancy with previous studies concerning the prognostic importance of nuclear E-cadherin in RCCs might be because of different study populations and follow-up times.

## Competing interests

The authors declare that they have no competing interests.

## Authors' contributions

HR carried out the immunohistochemical experiments and performed statistical analyses. HR, SK and PH evaluated the immunohistochemical staining and revised the manuscript. MHV participated in the design of the study and revised the manuscript. All authors read and approved the final manuscript.
